# Through 40,000 years of human presence in Southern Europe: the Italian case study

**DOI:** 10.1007/s00439-021-02328-6

**Published:** 2021-08-19

**Authors:** Serena Aneli, Matteo Caldon, Tina Saupe, Francesco Montinaro, Luca Pagani

**Affiliations:** 1 Department of Biology, University of Padova, Via Ugo Bassi, 58/B, 35131 Padova, Italy; 2 Estonian Biocentre, Institute of Genomics, University of Tartu, Riia 23b, 51010 Tartu, Estonia; 3 Department of Biology-Genetics, University of Bari, Via Edoardo Orabona 4, 70125 Bari, Italy

## Abstract

The Italian Peninsula, a natural pier across the Mediterranean Sea, witnessed intricate population events since the very beginning of the human occupation in Europe. In the last few years, an increasing number of modern and ancient genomes from the area have been published by the international research community. This genomic perspective started unveiling the relevance of Italy to understand the post-Last Glacial Maximum (LGM) re-peopling of Europe, the earlier phase of the Neolithic westward migrations, and its linking role between Eastern and Western Mediterranean areas after the Iron Age. However, many open questions are still waiting for more data to be addressed in full. With this review, we summarize the current knowledge emerging from the available ancient Italian individuals and, by re-analysing them all at once, we try to shed light on the avenues future research in the area should cover. In particular, open questions concern (1) the fate of pre-Villabruna Europeans and to what extent their genomic components were absorbed by the post-LGM hunter-gatherers; (2) the role of Sicily and Sardinia before LGM; (3) to what degree the documented genetic structure within the Early Neolithic settlers can be described as two separate migrations; (4) what are the population events behind the marked presence of an Iranian Neolithic-like component in Bronze Age and Iron Age Italian and Southern European samples.

**Supplementary Information:**

The online version contains supplementary material available at 10.1007/s00439-021-02328-6.

## Introduction

An increasingly detailed portrait of ancient Europe has been emerging in the last few years thanks to the joint analyses of archaeological findings, and the pattern of modern and ancient genetic variations elucidating past cultural transitions. The coarse edges of the European mosaic have been traced by three major contributions arriving in different periods and from different homelands: the first hunter-gatherers on the European ground, the Neolithic farmers from Anatolia, and the Bronze Age herders from the Steppe (Lazaridis et al. 2014; Haak et al. 2015). However, even though we have certainly found the tesserae composing the main figure, we still lack some fine details and we are far from putting together the blue sky pieces.

In this context, due to its position at the centre of the Mediterranean Basin, Italy is an ideal country to unearth the genetic footprints of at least some of the past demographic events in Southern Europe, thus offering the opportunity to add new pieces to the puzzle until clearer pictures emerge from our past. Indeed, its geographical features and the mild climate have been attracting people both near and far during the main stages of the peopling of the continent (Pais 1988).

Since Upper Palaeolithic, around 45,000 years ago (kya), modern humans have lived there (Benazzi et al. 2011, 2014), and during the LGM (18–20kya) Italy and the other Southern Mediterranean areas were used as refugia from the North (Stewart and Stringer 2012). The Italian Peninsula also played a major role in spreading the farming lifestyle, through at least two diffusion routes: one started from Apulia, where the most ancient findings associated with farming were discovered and following the Eastern coast reached the North, while the other started from East Sicily and travelled up along the Tyrrhenian coast (Pessina and Tiné 2008; Boattini et al. 2013).

During the Neolithic and for thousands of years later, the Mediterranean Sea itself contributed to shortening the distances and making Italy one of the gateways to the European continent: first acting for millennia as a barrier separating the African and the European continents, and then turned into a bridge as the first Bronze Age seafarers started to travel in open water (Broodbank 2006). Another hallmark of the Italian landscape contributed significantly to the cultural ferment of such times: the Alps. Indeed, they were so rich in copper that some Copper Age cultures arose in Italy, at Remedello and Rinaldone in the North and Gaudo in the South (Mallory et al. 1997). The diffusion of these metalworkers is evident in Ötzi, the South-Tyrolean Iceman. He carried a copper axe of the Remedello type, which was produced in Northern Italy using the ores of Tuscany (Keller et al. 2012; Artioli et al. 2017). During the Bronze Age, at least two ancestral sources moved through Italy, the Steppe-related component and an Iranian farmer-related component, whose amount steadily increased during the Iron Age period (Marcus et al. 2020; Fernandes et al. 2020). Moreover, the distribution of these components is still visible in the genomes of modern Italians (Sarno et al. 2017; Raveane et al. 2019).

Later on, the rise of the Roman civilization and its tight network of trade, political, religious, and cultural connections over one of the largest empires in the ancient world contributed to shuffle and mingle different genetic components across the Mediterranean, Continental Europe and Northern Africa (Antonio et al. 2019). With all this back and forth, the population inhabiting the country have been accumulating the genetic traces of these ancient wanderings, thus exhibiting the largest genetic diversity in Europe (Capocasa et al. 2014; Fiorito et al. 2016; Sazzini et al. 2016; Anagnostou et al. 2017; Raveane et al. 2019).

In the last few years, the scientific community has collected a huge amount of information from human remains and material culture discovered worldwide and through a wide range of time. Such data represent an extraordinary asset to solve the outstanding questions concerning the demographic events experienced by ancient populations. While a thorough assessment of all sources of scientific evidence would be the preferred approach to tackle such a broad research question, here we will only focus on genetic evidence as the primary source of information.

**Fig. 1:**
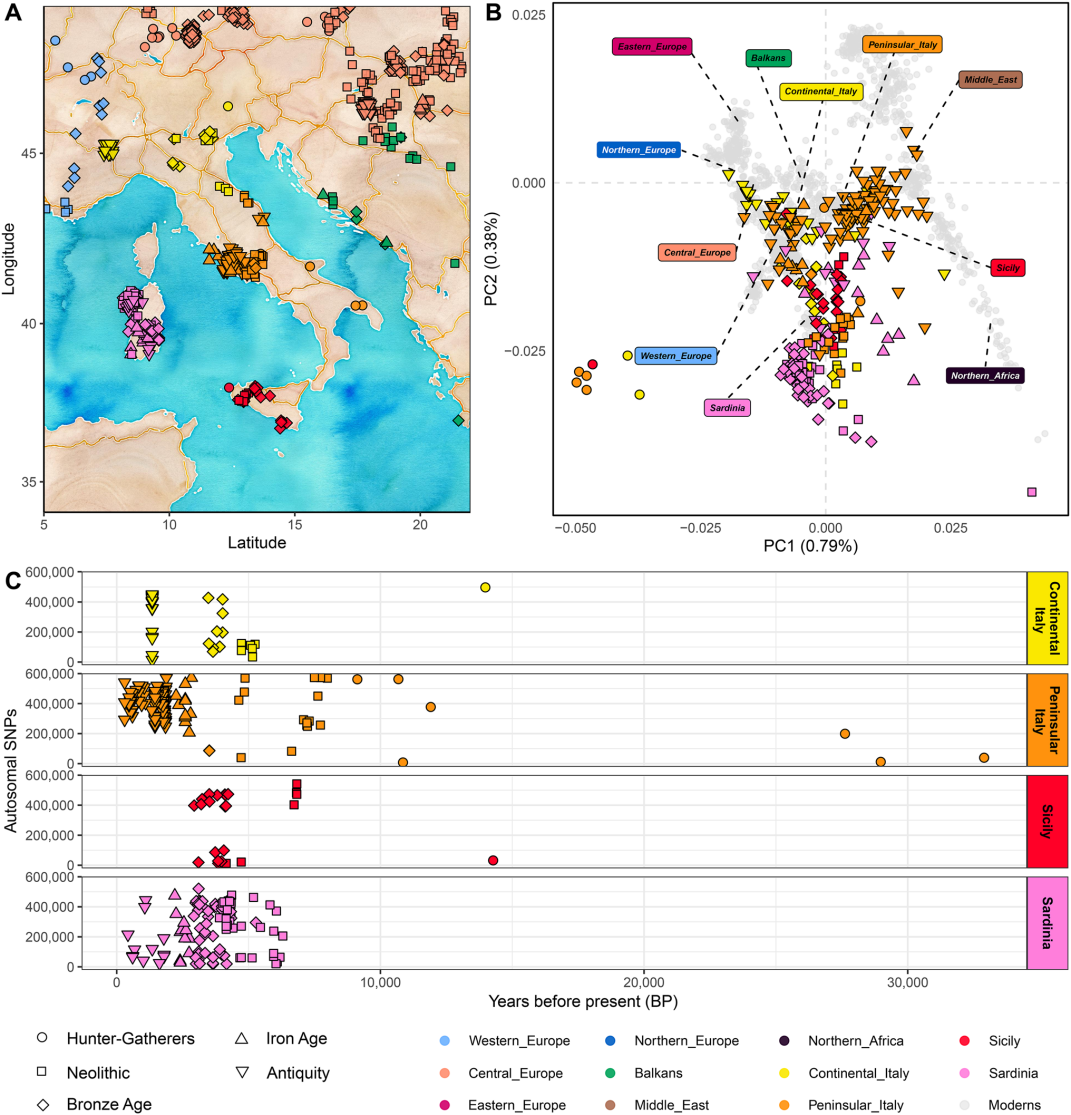
Ancient Italian individuals discussed in this review. **A** Geographical location of ancient individuals from Italy and surrounding areas. The Italian ancient individuals are divided into four main categories: Continental Italy (yellow), Peninsular Italy (orange), Sicily (red), and Sardinia (pink); **B** Principal component analysis (PCA) projecting the ancient Italian individuals onto the genetic variation of present-day individuals (in grey). **C** Chronological distribution (*x*-axis) and the number of autosomal SNPs of the ancient Italian individuals (*y*-axis, proxied by the number of available SNPs from the standard 1240K+HO (Human Origin array) SNP set)

In this review, we collected and analysed all the genetic data of ancient Eurasian individuals which have been generated so far, focusing on their variable sites and clustering ancient samples by the culture of major genetic components with no assumptions on their belonging to discrete population units, together with a reference of modern populations from the same areas (Fig. 1, Supplementary Figs. 1, 2 and Supplementary Table 1). In particular, more than three hundred of them come from Italy, ranging from the Late Palaeolithic to Medieval times, thus offering the exciting opportunity to glimpse some broader pictures from our past. For this reason, taking Italy as a case study we recapitulate the main steps of the peopling of Europe, highlighting outstanding questions that may hopefully be addressed in the coming years.

### From the first peopling of Europe to the Villabruna hunter-gatherers

The first humans inhabiting Italy were archaic humans: remains of putative *Homo erectus* (Peretto 2006), *Homo heidelbergensis*/Ceprano and Neanderthal were found throughout Italy and date back from as early as 1 million years ago to ~ 40kya (Manzi et al. 2001; Bruner and Manzi 2006; Lari et al. 2015). The *Homo sapiens* occupation of the Italian Peninsula started quite early and intriguingly overlaps the Neanderthal one. Notably, the Grotta del Cavallo (Apulia, Peninsular Italy) (Benazzi et al. 2011) provides one of the earliest evidences of sapiens in Europe and it is associated with a cultural package, the Uluzzian technology, which may show influences from both human groups. Ancient genomic evidence (Fu et al. 2016) showed that the Italian genetic substrate was connected with that of continental Europe, with remains found in archaeological sites like Paglicci and Ostuni (both located in Apulia and dating back to 33kya and 28kya, respectively) showing genetic affinities with the so-called “Vestonice cluster”. This group of individuals spanned all the way from the Russian Steppe to Central Europe until the beginning of the last Ice Age (25–20kya) and falls outside of the human diversity described by modern human samples [Supplementary Fig. 3, in some cases their high genetic diversity when compared to present-day human populations is reflected in the Principal Component Analysis (PCA) by a shift towards the center of the coordinate system (Fu et al. 2016)]. These Italian sites are all associated with Gravettian material culture (33-21kya), which is argued to have provided a substrate for the in-situ development of Early Epigravettian, the landmark of the fossil record dating to the Last Glacial Maximum (LGM) and potentially pointing to the role of Central and Southern Italy as glacial refugia (Stewart and Stringer 2012).

**Fig. 2:**
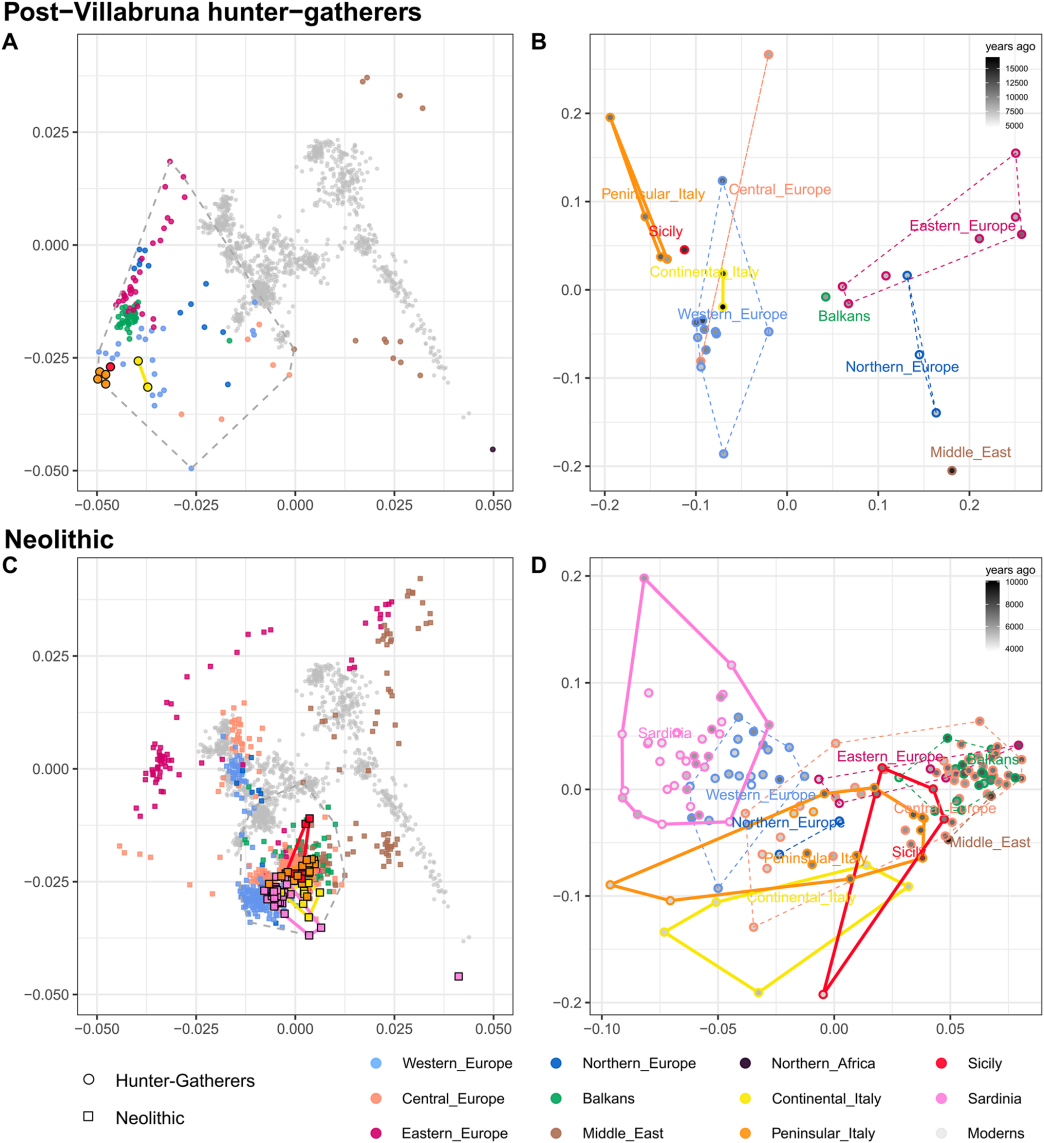
PCA and outgroup *f*_3_ multidimensional scaling (MDS) for samples dated back to Paleo-Mesolithic (hunter-gatherers) and Neolithic periods (**A**–**D**, respectively). Samples were divided into four groups according to their chronological and cultural affiliation and projected onto the genetic variation of modern West Eurasians (left column, panels **A**, **C**) along with a number of modern reference samples from neighbouring regions. Samples within the dashed grey polygons were also used to construct a pairwise matrix of outgroup *f*_3_ distances in the form of *f*_3_ (Mbuti; X, Y) on which an MDS was performed, hence providing a complementary visualization to the PCA. The chronological dating, based on mean date BP (before present), was used to color-code the samples (right column, panels **B**, **D**)

During the LGM, at around 20-19kya, Italy was by and large disconnected from the rest of continental Europe by the Alpine Icecap (Peresani et al. 2020). At the same time, it was accessible from the Balkan Peninsula through land bridges spanning the Adriatic Sea. These connections provided crucial for the establishment of trade and cultural exchanges with groups from the Balkans and the Black Sea region (Peresani et al. 2020), which ultimately resulted in genetic exchanges. The most notable evidence of such a gene flow is represented by the so-called Villabruna replacement, which is represented by the arrival of genetic components with a higher affinity to contemporary Near Eastern groups and which formed the basis for the post-Ice Age European genetic landscape (Fu et al. 2016). The earliest evidence of such a massive replacement is represented by the Tagliente2 sample from Riparo Tagliente (near Verona, Northern Italy) dated to 17kya and for which a whole-genome shotgun sequence is available (Bortolini et al. 2021). Although based on mtDNA evidence alone, earlier traces of Villabruna-like genome may be seen in some Paglicci samples dated to 19kya, at the very end of LGM (Posth et al. 2016). Villabruna and Tagliente2 (in yellow, Fig. 2A, B) fall at the centre of the other post-Villabruna HG samples, from which they differentiate towards Italy and the rest of Europe (Fig. 2B), showing the pivotal role of these early Italian samples in explaining the subsequent Western Hunter Gatherers (WHG) genetic diversity. The emerging scenario hence seems to place Italy as the first step of the re-colonization of post-Ice Age Europe by settlers coming from the East potentially as early as 19 or 20kya. This population movement introduced in Southern Europe, through demic diffusion, the Epigravettian cultural package (Bortolini et al. 2021) and may have initiated other cultural transitions given the presence of a similar genetic component found in the genome of the individual ElMiron, who lived ~ 18.5kya in Spain and that was associated to Magdalenian culture (Fu et al. 2016). A similar, parallel movement from the Balkan/Black Sea region towards the North, with genetic contributions from Central/East Eurasia, may explain the origin of Eastern Hunter Gatherers (EHG), who indeed seem to align along an orthogonal axis from the one described by the WHG (Fig. 2B), although this is beyond the scope of the current review. While LGM can be seen as the major cause for the disappearance of pre-Villabruna people in Central and Northern Europe, it is still poorly understood what may have caused the disappearance of the genetic components related to the Vestonice cluster in later individuals in Italy. One scenario may involve the Epigravettian package brought by the Villabruna people as the key for their ultimate success, although more Southern Italian samples from 20 to 15kya may show a less dramatic scenario, perhaps providing a clue to interpreting the so-called WHG component in light of pre and post Villabruna admixtures (Sazzini et al. 2020). Another outstanding question is the role of the major Italian islands (Sicily and Sardinia) during the pre and post-Villabruna periods. We know from Grotta d’Oriente (Sicily) that WHG arrived there at least as early as 14kya (Catalano et al. 2020). However, the presence of Vestonice-like individuals in Sicily and of Vestonice or Villabruna-like individuals in Sardinia is an as-yet unexplored chapter.

### Neolithic and copper age

The term “Neolithic Revolution” refers to the five millennia-long process (approximately between 12,000 and 7000 ya) involving dramatic changes in the behavior, culture, and ecology of human populations, culminating in the domestication of plants and animals, and the almost ubiquitous adoption of pottery (Fowler et al. 2015). The Western Eurasian origin of these changes has been geographically placed in the Fertile Crescent, an area including the Southern part of Anatolia, modern-day Western Iran, Iraq, Syria, Lebanon, Israel, Palestine, Jordan, and Egypt. After their emergence, techniques and artifacts associated with Neolithic culture spread very fast, reaching Iberia already by ~ 7450 ya (Hofmanová et al. 2016). The how and when of its expansion in Europe has been a very debated subject till the last decades, in which a spectrum of purely demic/purely cultural models was advocated by many scholars, including geneticists (Ammerman and Cavalli-Sforza 1984). Although many genetic studies, including the pioneering ones started by Cavalli Sforza and colleagues, tentatively attempted to address the Neolithic Revolution dynamics (Ammerman and Cavalli-Sforza 1984; Cavalli-Sforza 2001), the ultimate verdict was reached only recently, when the analysis of genetic material extracted from human remains confirmed a mostly demic diffusion, with a complex genetic interaction with local hunter gatherers (Skoglund et al. 2012; Gamba et al. 2014; Lazaridis et al. 2014; Olalde et al. 2015). It is globally accepted that this demic diffusion followed both a Mediterranean route (following the Mediterranean coasts, including Italy) and a continental route (Balkan route reaching Central and Western Europe through the Danubian valley), with the terminal fringes of this migration admixing again. Furthermore, quantitative and systematic analysis of the archaeological records found support for an expansion characterised by “boom and bust” (Shennan et al. 2013), in which expansions were followed by stasis. Archaeological records, such as the Impressed/Cardial Ware (Southern Europe along the Mediterranean shores) and Linear ceramic cultures (LBK, Central Europe) named after the peculiar practice of pottery ornamentations, suggest the existence of at least two different routes of colonisation of Europe. Although the global outlines of the European Neolithization are now known, many specific and local aspects are missing. As an example, despite their marked difference in Archaeology, it is not clear how these groups from the two waves were differentiated.

Although in two expanding populations from the same source or geographic area, the amount of genetic drift would be very low, investigations on modern samples have shown that the state of art genetic analysis would be able to pinpoint even the subtlest difference among different groups (Pankratov et al. 2020).

However, clear genetic signals of this double expansion have not been found so far (Haak et al. 2015). This could possibly depend on the small sample size of the available ancient populations, combined with the relatively low quality of genotype and sequence data, not having enough power to recover the subtle differences between the two Neolithic founder populations. However, with the increase of ancient samples examined, subtle differences between Central-West and Central-East Europe started to emerge, with samples from Western and Eastern Europe forming two distinct clusters in the PCA (Fig. 2C) (Olalde et al. 2015; Rivollat et al. 2020; Marcus et al. 2020; Saupe et al. 2021). *F*4 statistics and related method (qpAdm) showed that this separation is possibly due to different amounts of ancestry related to WHG. It may be possible that the populations spreading through Central and Mediterranean routes mixed differently with residing hunter-gatherer populations, with the former witnessing a higher number of admixture events, in line with some archaeological evidence suggesting a sparser mesolithic hunter-gatherer population in Southern Italy, although new archaeological surveys are needed (Mussi 2006; Martini 2019). On the other hand, the genetic drift and founder effect acting independently in the two groups might have given origin to differences in allele frequencies and haplotype patterns, which have not been uncovered so far. Notably, when focusing on Italy, most Italian Neolithic samples are closely related to the Central European cluster (Fig. 2C, D, salmon color), with the only exception of Peninsular Chalcolithic (6632–4630 ya) and Neolithic Sardinian (6299–3896 ya) individuals (Fig. 2D, pink and orange circles) which are more related to the Mediterranean cluster. This peculiar pattern is potentially mirrored by Y-chromosome analyses (Rohrlach et al. 2021), an adequate coverage of which is beyond the scope of the current review.

Nevertheless, many aspects of the Neolithic diffusion West of the Adriatic have not yet been characterised. The lack of Neolithic samples from Peninsular Southern Italy, the first Italian area to be affected by the Neolithic wave, makes it impossible to understand the most probable genetic sources. Another open question concerns whether multiple streams reached Italy, as cultural differences in Sicilian and Adriatic Italy (e.g., Apulia) tentatively suggest (Pessina and Tiné 2008).

### Bronze age

European populations were just swept up by the Neolithic wave that another time of great demographic and cultural turmoil was approaching. The Eurasian Bronze Age (between around 5000–3000 ya) was a period of major cultural changes, indeed, with the introduction of new metalworking techniques and breakthrough innovations, such as the wheel, the chariot and the ox-drawn plough, the rising of the first cities and the intensification of commercial networks whose routes for selling metal goods connected people from far and wide (Harding and Fokkens 2013; Allentoft et al. 2015). However, whether these changes were gradually diffused by the movement of people or the spreading of ideas remained disputed for a while.

The initial answers to this debate came from the Pontic-Caspian steppes, where the Yamnaya, a nomadic culture based on sheep and cattle herding, emerged at least 5000 ya. The *kurgan*, a round tumulus or barrow built upon a grave, was one of their distinctive elements, representing also an important step in the direction of modern civilization because, for the first time, single rather than collective graves were introduced (Manco 2016). Gradually, elements of their culture began to be found in Europe and Central Asia, up to the foothills of the Altai Mountains. From the genetic point of view, the Yamnaya population was a mixture of different ancestries: EHG and CHG-related ancestry, the latter possibly deriving from the Maykop culture (Jones et al. 2015), as well as traces of the first farmers of Northern Iran, which together are usually referred as “Steppe-related ancestry”. Steppe-related ancestry appeared in Europe with the Corded Ware population as early as 4750 ya (Allentoft et al. 2015; Haak et al. 2015). Along with part of their DNA, the Corded Ware shared with the Yamnaya many other traits, such as the large burial mounds, the intensive use of horse and herding, a male-centred culture and the finely executed copper axes (Haak et al. 2015). Another European society shared some cultural as well as genetic legacy from the Yamnaya—the Bell Beakers—but, in contrast to the Corded Ware (Saag et al. 2017; Mittnik et al. 2018), they exhibited a high genetic heterogeneity: while Iberian Bell Beakers were genetically indistinguishable from the people who lived there earlier (Olalde et al. 2018), Bell Beakers from Central Europe showed a considerable amount of their ancestry deriving from the Steppe populations. With the Steppe-related ancestry, the third ancestral component of the European genetic make-up and the one contributing the most to it had been found (Lazaridis et al. 2014). At that point, the European genetic puzzle was apparently completed with the Corded Ware and Bell Beaker migrations as the major spreaders of the high level of Steppe-related ancestry we see in modern-day Europeans, at least in Northern and Central Europe (Raveane et al. 2019).

Southeastern Europe was the first European region to receive the Steppe-related ancestry, with sporadic individuals showing this component in Bulgaria as early as 6700–6500 ya (Mathieson et al. 2018), to the point that during the Bronze Age almost everyone there harboured around 30% of Steppe-related ancestry. Also, Sicily received this ancestry after ~ 4150 ya, while Sardinia had to wait until the Iron Age (Sarno et al. 2017; Fernandes et al. 2020). More specifically, the timing of the Steppe-related ancestry arrival in Italy was a central topic of a recent study, examining human remains from Northeastern and Central Italy dated to the Chalcolithic and Bronze Age (Saupe et al. 2021). As a confirmation of other Central European studies (Olalde et al. 2018), they found a gradual increase in such ancestry over time, with the first appearance in Early Bronze Age Italians (Italian Bell Beaker ~ 4000 ya (Olalde et al. 2018), Italian Remedello ~ 3900 ya (Allentoft et al. 2015), and Grottina dei Covoloni del Broion dated ~ 3800 (Saupe et al. 2021). Moreover, Saupe and colleagues retraced the arrival of the Steppe-related ancestry in Northeastern Italy as early as 3900 ya and in Central Italy as early as 3,550 ya.

The Bronze Age Aegean civilizations, mainly represented by Minoans and Mycenaeans, also harboured up to a quarter of their ancestry ultimately connected with ancient populations from Caucasus and Iran. Nonetheless, only the Mycenaean individuals also showed the EHG-related component (Lazaridis et al. 2017). For this reason, at least in the Minoans, the three-way split ancestral contribution is not completely fulfilled and raises other issues about who brought the Caucasus/Iran-related component into Europe.

The widespread commercial networks, facilitated by the introduction of chariots and the technological advances in seafaring, allowed long-distance mobility never seen before (Abulafia 2011). In this context, the geographical position of Italy at the centre of the Mediterranean Sea could help reconcile the cryptic source of the Caucasus-related ancestry, and several aDNA studies based on Italian archaeological sites came out in the last few years also trying to address this issue (Antonio et al. 2019; Marcus et al. 2020; Fernandes et al. 2020). The work from Antonio and colleagues examined the genetic transitions experienced by Central Italians which, during the Iron Age, would have given origin to the founders of the Roman civilization (Antonio et al. 2019). While they detected the suggestive presence of a small amount of the Caucasus/Iran-related component as early as the Neolithic period, they observed an increase of that component during the Bronze and Iron Age (between ~ 4800 and 2850 ya), possibly due to increased trade-driven mobility.

**Fig. 3:**
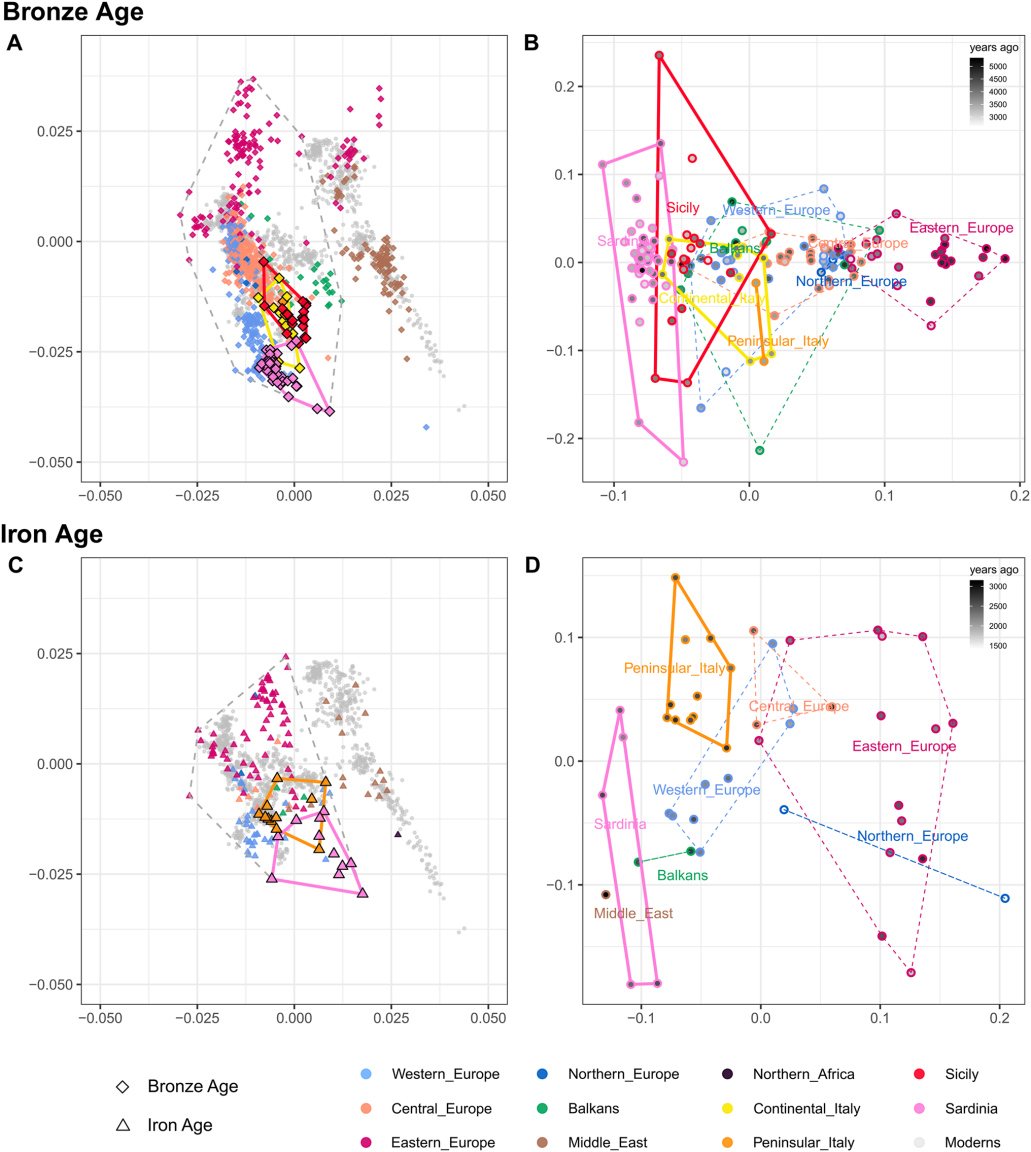
PCA and outgroup *f*_3_ multidimensional scaling (MDS) for Bronze and Iron Age samples (**A**–**D**, respectively). Samples were divided into four groups according to their chronological and cultural affiliation and projected onto the genetic variation of modern West Eurasians (left column, panels **A**, **C**) along with a number of modern reference samples from neighbouring regions. Samples within the dashed grey polygons were also used to construct a pairwise matrix of outgroup *f*_3_ distances in the form of *f*_3_ (Mbuti; X, Y) on which an MDS was performed, hence providing a complementary visualization to the PCA. The chronological dating, based on mean date BP (before present), was used to color-code the samples (right column, panels **B**, **D**)

The same long-distance mobility with the resultant genetic transitions has been highlighted by the other recent studies focusing on the islands of the Western Mediterranean Sea: the Balearic Islands, Sardinia, and Sicily (Marcus et al. 2020; Fernandes et al. 2020). Both Sardinia and Sicily received the cultural influxes of the Bell Beaker complex after ~ 4450 ya (Ugas 2006; Harding and Fokkens 2013; Fernandes et al. 2020), but similarly to the Iberian situation, that happened without bringing along the Steppe-related ancestry: indeed, Beaker-associated individuals in both islands do not harbour Steppe ancestry (Olalde et al. 2018; Fernandes et al. 2020). The Steppe ancestry started appearing in Sicily around 4150 ya, during the Early Bronze Age, as demonstrated by qpAdm modelling on autosomal genetic variation with two notable outliers carrying up to 20% and 40% of this genetic component (Sicily_EBA8561 and Sicily_EBA11443, (Fernandes et al. 2020). Starting from the Middle Bronze Age (3750–3450 ya), Sicilian samples show a considerable shift in the PCA towards Minoans and Mycenaeans (Fig. 3A, Minoans and Mycenaeans are grouped in the Balkan area in green) and can be modelled with the Iranian-related component in a percentage around 15% (Fernandes et al. 2020). This data opens up the fascinating possibility that this mysterious ancestry must have reached Southern Italy before the occupation of the southern coastal areas of Italy (Magna Graecia). Conversely, ancient Sardinians showed a higher degree of genetic continuity from the Neolithic to the Bronze Age, with almost all individuals showing similar proportions of Anatolian Neolithic and WHG ancestries. This peculiar situation strongly differs from many other European regions, where the Bronze Age brought huge demographic and cultural turmoil and suggests genetic isolation from mainland populations (Marcus et al. 2020). Two interesting exceptions within the Sardinian individuals regard two outliers showing the former a huge proportion of Northern African ancestry (around 77%) and the latter carrying Eastern Mediterranean or Steppe-related components (Fernandes et al. 2020). During the Middle Bronze Age, about 3550 ya, archaeological evidence showed the appearance of the Nuragic culture, named after the Sardinian most characteristic stone towers called *nuraghi*. Despite the absence of the Iranian-related ancestry until the Iron Age, this period was characterized by the intensification of trades and cultural exchanges bridging together the lands facing the Mediterranean, as testified, for instance, by the arrival of copper goods from Cyprus in the Late Bronze Age Sardinia (Sabatini and Lo Schiavo 2020) and the presence on the island of Mycenaean, Levantine and Cypriot traders. Nevertheless, evidence of gene flows altering the genetic continuity did not appear until after the Nuragic period (Marcus et al. 2020).

### Iron age

The Iron Age, the latest protohistoric period before the beginning of Antiquity History and Historiography, in Italy and Western Europe started between the 2nd and the 1st millennium BCE (Before Common Era) and ended with the Roman conquest (Peroni 1989; Cunliffe 2008). This period was characterised by the development of new metallurgic techniques and the consequent mass appearance of iron artifacts in archaeological sites (Bietti Sestieri 2018). It was also a period of demographic growth, social, and political stratification as well as the intensification of trade networks. Multiple Iron Age populations characterized by different languages and cultures lived in Italy but, genetically speaking, they started to approximate the modern Italians (Aneli et al. 2021; Antonio et al. 2019).

The most particular case is represented by the Sardinians: a strong Anatolian Neolithic footprint is visible in the Iron Age samples due to the persistent isolation in previous times and despite the documented contacts with Mycenaeans and Phoenicians (Marcus et al. 2020). Phoenician presence has been dated to as early as the late ninth century BCE (Moscati 2005). Indeed, Eastern Mediterranean genetic signatures have been only found among the post-Nuragic individuals in Phoenician/Punic sites, which could be modelled as direct immigrants or close relatives of the newcomers. Moreover, also the diffusion of Steppe and Iranian-related ancestries, as well as a Northern African component that likely arrived during the Punic (Chartaginean) domination (found in individuals from the Punic Villamar site), has been highlighted in Iron Age samples (Marcus et al. 2020).

As Sardinia, also Sicily was an important Punic trading post, especially on the western coast. Despite the paucity of samples from this area, the presence of Northern African ancestry on the island could be tentatively reconducted to the Iron Age (or at least Antiquity), because, although absent in previous time layers, it’s present in modern Sicilians (Marcus et al. 2020; Fernandes et al. 2020). In addition to the Punics, the other main shapers of the Iron Age Sicilian genetic make-up were the Greeks, who established colonies on the island (and South Italy) starting from the eighth century BCE (Abulafia 2011; Sarno et al. 2017). According to recent findings, the Greek colonization was a more gradual and peaceful process than that described by ancient historians, characterized by greater interaction and close cohabitation between newcomers and local people (Rathmann et al. 2019). This is probably the reason why modern southern Italians display a varying amount of Greek genetic influence (Sarno et al. 2017), which could be described as a “Mediterranean continuum”. The Greek-speaking communities in South Italy (Grecani in Calabria and Griko in Apulia) are clearly placed in this *continuum* and, although there may have been a more recent migration during the Byzantine times (Early Middle Ages), those Greek-speaking communities are believed to have lived in Southern Italy probably since the Magna Graecia times.

If on the one hand Phoenician and Greek Mediterranean-wide trade networks left genetic traces in Sardinians and Southern Italians, on the other hand, it surely had a cultural impact on Etruscans, a population who lived in the Central Italian area named Etruria (between Lazio, Umbria and Tuscany). The Etruscan civilization and, specifically its earliest phase called Villanovan culture, dates back to the ninth century BCE and ended with the slow and gradual Roman assimilation from the sixth century BCE. However, where the Etruscans came from is still uncertain. Both ancient and modern historians have different opinions on the matter and, while some researchers believe in an autochthonous process of formation from the previous Villanovan society, others claim an Anatolian origin (Achilli et al. 2007; Tassi et al. 2013). For instance, a multi-step origin explaining the modern Tuscans genetic composition has been proposed: a proto-Etruscan population, born in a Southeastern region of the Middle East, would have migrated to the Caucasus, then to Lydia and finally arrived in Central Italy at the beginning of Iron Age (Pardo-Seco et al. 2014). However, it is clear that more genetics studies are needed to shed light on this disputed matter.

During the later Iron Age, Etruscans were replaced in their political influence on Central Italy by Rome’s rule, a new town that arose in Latium (Central Peninsular Italy) on the banks of the Tiber river. Central Italy and Rome inhabitants of the first millennium BCE exhibited high genetic variability, in particular, they showed a relevant Steppe-related ancestry, an increase in the Iranian-Neolithic component, respect to previous times, and the appearance of the first Northern African signatures on the Peninsula (Antonio et al. 2019). The appearance of such different contributions is the main consequence of the great mobility of people, which increased even more in the later stages of Rome’s history during the Republic (509-27 BCE) and the following Empire periods (27 BCE—476 CE; CE: Common Era). During the Iron Age and the first stages of Antiquity, long-distance mobility was promoted by the cosmopolitan nature of the Roman Empire and people from far and wide arrived in Rome, creating a melting pot of languages, cultures, and genes (Abulafia 2011). In particular, many people came from the East (Greece, Syria, Egypt), the richest and most densely inhabited region of the Empire, thus resulting in a genetic shift towards the Eastern Mediterranean areas in individuals from this period (Iron Age) and the subsequent imperial period—classified here as Antiquity (Fig. 3C and Supplementary Fig. 4A). Figure 3C, D clearly points toward the high genetic heterogeneity within Roman and Central Italian individuals, which is a direct consequence of the key role of Rome as the geographical, cultural, and political crossroad of Eastern and Western Mediterranean. These figures also show that the genetic make-up of Italy during the Iron Age was close to the pattern of modern-day populations.

### Antiquity and the making of modern Italians

From the third century CE, the Roman Empire went into a deep crisis due to different causes like strong political instability and military anarchy, population decrease (wars and epidemics), economic stagnation, and invasions from non-Roman populations (Harper 2017). The Empire was also divided into two: the Eastern Roman Empire with the capital Byzantium (today Istanbul, Turkey) and the Western Roman Empire with capital Rome. The latter, poorer in resources and weaker, fell definitively in 476 CE with the deposition of the last Roman emperor by the barbarian commander Odoacer (Mazzarino 1988).

The split of the Empire also had repercussions on the Italian genetic composition. Antonio and colleagues observed a shift from the Eastern Mediterranean to continental Europe in Central Italian samples (Supplementary Fig. 4), which probably resulted from the reduction of the East–West mobility, the demographic drop, and the arrival of people from Central and Eastern Europe. Besides the Romans, the Longobards were another relevant population for the Italian cultural landscape during the Late Antiquity. Samples from the Longobard cemetery of Collegno (Piedmont, Continental Italy) show genetic affinities to Bronze Age people from Central and Eastern Europe (Amorim et al. 2018). However, the genomic background of Northern Italian people preceding the Longobard arrival is still unknown. Thus, it is unclear if they contributed to diffusing the Central European genetic component and what was their real genetic contribution to the Italian inhabitants. Altogether, with the end of the Antiquity and the beginning of the Middle Ages (fifth century CE), the Italian genomic scenario reached most of the modern time composition with a high level of individual variability.

**Fig. 4:**
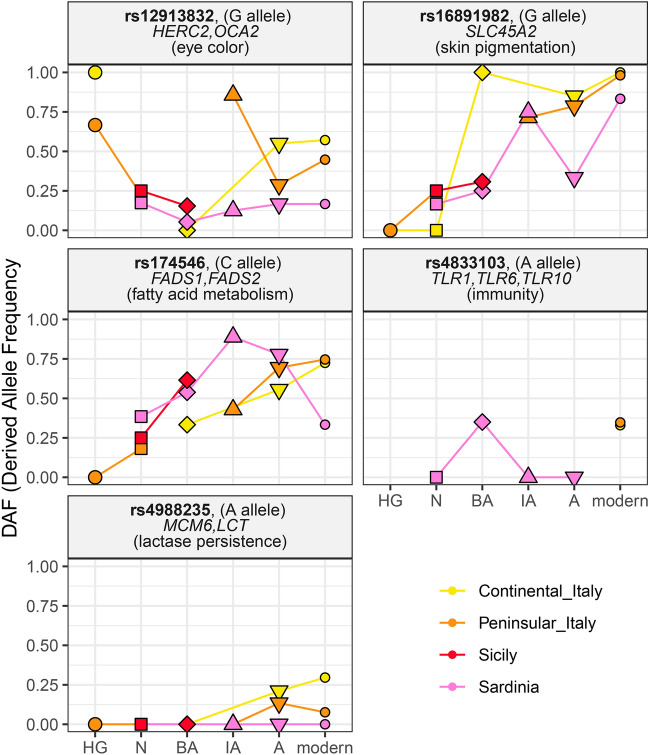
Derived allele frequencies across the four Italian macro-areas for five genetic variants reported to be the strongest significant signals of selection in (Mathieson et al. 2015). The name of the variant (in bold), the allele for which the genetic frequencies are computed (the derived one), the name of the gene, and the associated function are shown above each plot

The stratified contribution of each time layer to contemporary allele frequencies is evident in Fig. 4, where we magnify the time-dependent allele frequency changes for five variants which have been reported to be genome-wide significant signals of selection among the three main ancestries composing the modern European genetic make-up—hunter-gatherer, early farmer and Steppe people (Mathieson et al. 2015). The variants in Fig. 4 are associated with lactose metabolism, immune system, skin pigmentation, and eye color (Enattah et al. 2002; Bersaglieri et al. 2004; Soejima and Koda 2007; Sturm et al. 2008; Eiberg et al. 2008; Barreiro et al. 2009; Wong et al. 2010; Teslovich et al. 2010; Divangahi 2013). However, the fact that little has changed after Iron Age and Antiquity does not mean that movements of people across Europe and Italy would have come to an end after that time, rather the incoming populations would have not been so highly diverse as they were before (Günther and Jakobsson 2016).

**Fig. 5:**
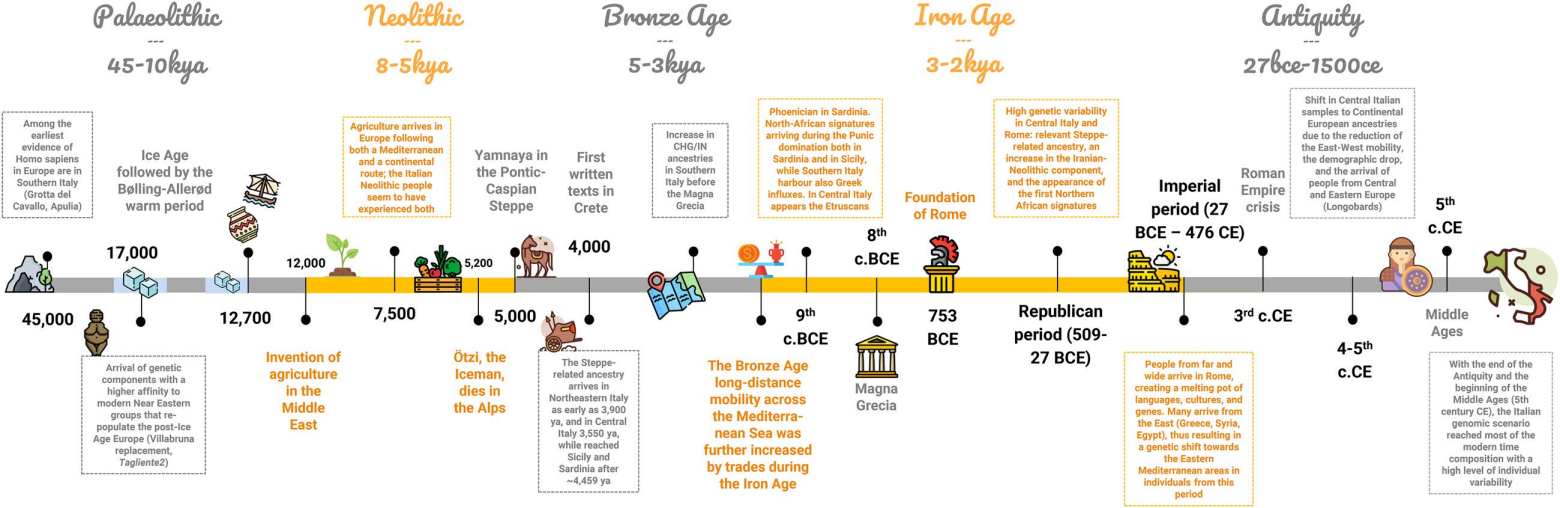
Timeline summarizing the main demographic events experienced by the ancient Italian populations from the Palaeolithic to the Middle Ages. The time range for each period is relative to Italy or, more generally, to Southern Europe

Homogenizing effects introduced by the pervasive Neolithic and Bronze Age demic diffusions, paired with a steady post-Neolithic population growth that made local populations less and less prone to major genetic turnovers, established a strong genetic continuum all across West Eurasia, making geographic distance the best predictor of similarity and dissimilarity between populations (Pagani et al. 2016). Therefore, contemporary Italians can be seen as the inhabitants of a pier stretched across the Mediterranean Sea, whose genome has been shaped by millennia of landings that made it resonate with the ones of the neighboring populations ashore (Fig. 5).

## Materials and methods

### Datasets

We collected all available ancient human DNA data from the literature, covering a period from Upper Palaeolithic to historical times and mostly coming from Western Eurasia.

Specifically, we downloaded the 1240K+HO dataset from Dr David Reich laboratory, which comprises present-day and ancient DNA data (version V42.4; https://reich.hms.harvard.edu/allen-ancient-dna-resource-aadr-downloadable-genotypes-present-day-and-ancient-dna-data (Patterson et al. 2012; Gamba et al. 2014; Olalde et al. 2014,2015, 2018, 2019; Skoglund et al. 2014; Lazaridis et al. 2014, 2016, 2017; Allentoft et al. 2015; Fu et al. 2015, 2016; Günther et al. 2015; Jones et al. 2015, 2017; Mathieson et al. 2015, 2018; Cassidy et al. 2016; Martiniano et al. 2016, 2017; Schiffels et al. 2016; Omrak et al. 2016; Hofmanová et al. 2016; Broushaki et al. 2016; Kılınç et al. 2016; González-Fortes et al. 2017, 2019; Schuenemann et al. 2017; Unterländer et al. 2017; Saag et al. 2017, 2019; Haber et al. 2017, 2019; Sikora et al. 2017, 2019; Lipson et al. 2017; Damgaard et al. 2018a, b; Fernandes et al. 2018; Krzewińska et al. 2018a, b; Mittnik et al. 2018, 2019; van Loosdrecht et al. 2018; Amorim et al. 2018; Harney et al. 2018; Feldman et al. 2019a, b; Järve et al. 2019; Sánchez-Quinto et al. 2019; Schroeder et al. 2019; Wang et al. 2019; Villalba-Mouco et al. 2019; Biagini et al. 2019; Brace et al. 2019; Jeong et al. 2019; Narasimhan et al. 2019; Antonio et al. 2019). In addition, we added: (1) Bronze Age Southern Levant DNA data (Agranat-Tamir et al. 2020); (2) ancient DNA data from the Balearic Islands, Sicily, and Sardinia (Fernandes et al. 2020); (3) ancient samples from Sardinia spanning the Middle Neolithic to present days (Marcus et al. 2020); (4) the Italian Chalcolithic and Bronze Age samples (Saupe et al. 2021); (5) the genotypes of an Italian hunter-gatherer from the Late Epigravettian site of Riparo Tagliente dated around 17kya (Bortolini et al. 2021).

Finally, we also recovered additional modern-day Italian samples (Raveane et al. 2019).

We converted the dataset to the PLINK format using *convertf* (for the dataset in EIGENSTRAT format) and the software PLINK 1.9 (Chang et al. 2015) for the vcf file of (Marcus et al. 2020).

We extracted only those modern and ancient samples from the 1240K+HO coming from Western Eurasian countries. More specifically, we selected ancient samples located at latitudes higher than 22 and longitudes between − 15 (Canary Islands) and 60 (the political border between Iran and Afghanistan). We kept the Mbuti individuals from Congo [HGDP, (Patterson et al. 2012)]. We removed items with the string “*Ignore*” in their “Group.Label” column of the*.anno* file. Finally, we removed those whose “Assessment” column did not contain the string “*PASS*”. If there were duplicates among ancient individuals, we selected the sample with the highest number of SNPs hit on autosomal targets.

To further refine the backbone of the modern-day European genetic variability, without mixing different genotyping techniques, we selected the modern samples flagged as “*PASS (genotyping)*” (“Assessment” column) and we removed those coming from Uzbekistan, Kazakhstan, Algeria, Morocco, Tunisia, Libya, as well as some populations from Russia. We excluded SNPs on sexual chromosomes and those monomorphic or with more than 5% missing data in the modern samples using PLINK.

We extracted the bulk of variants built on modern samples from each dataset and we merged them with PLINK. Finally, we excluded the ancient samples with more than 5000 missing SNPs and we converted the dataset to the EIGENSTRAT format using *convertf.*

Our final dataset contained 2144 ancient and 1091 modern samples (Supplementary Tables 1, 2).

We assigned each sample to a geographic macro-area among “Northern Europe”, “Western Europe”, “Eastern Europe”, “Central Europe”, “Continental Italy”, “Peninsular Italy”, “Sicily”, “Sardinia”, “Balkans”, “Middle East” and “Northern Africa” and to a period among “Hunter-gatherers”, “Neolithic”, “Bronze Age”, “Iron Age”, and “Antiquity” based on the available cultural information (Supplementary Fig. 1A and Supplementary Fig. 2). Given the complete lack of ancient data from Corsica, we do not discuss the genetics of Corsica in this review, even though its geographic proximity to Sardinia and the Italian mainland suggests that many of the demographic changes of Italy would be shared with the island.

We additionally distinguished, among the hunter-gatherers, the Pre- and Post-Villabruna samples using 18kya as a threshold.

### Derived allele frequencies variability of selected variants

We computed the derived allele frequencies for the twelve genetic variants, which have been found under selection among hunter-gatherers, early farmers, and steppe ancestry (Mathieson et al. 2015). Given that some of these SNPs were missing in the Human Origin arrays, we used the 1240K dataset (V42.4) which contains the same ancient samples of the 1240K+HO and less modern samples (Supplementary Table 3 contains the list of modern samples of the 1240K dataset) (1000 Genomes Project Consortium et al. (1000 Genomes Project Consortium et al. 2015).

### Principal components analysis

We performed principal component analysis using the *smartpca* function implemented in EIGENSOFT software 8.0.0 (Patterson et al. 2006). Specifically, we projected the ancient samples’ genotypes onto the principal components inferred from the modern individuals’ genetic variability. We used the options “*lsqproject*” and “*shrinkmode*”.

### *f*_3_-statistics

To explore the genetic affinities among ancient samples, we performed a series of Outgroup *f*_3_ (Patterson et al. 2012; Raghavan et al. 2014) in the form (X, Y; outgroup: Mbuti) using the qp3Pop function (version 650) implemented in ADMIXTOOLS (Patterson et al. 2012). Since the Mbuti population is an outlier with respect to the samples analysed in this review, the resultant *f*_3_ measures the shared drift between X and Y (i.e., the common branch length from the outgroup), in a way that the higher is the *f*_3_, the closer is the relatedness between the two populations.

We grouped the non-Italian ancient samples into higher cultural labels, which are shown in the column “Higher.Label” of Supplementary Table 1 and we performed the Outgroup *f*_3_, after removing samples flagged as outliers or with family relationships with other individuals (Supplementary Table 4).

Finally, we computed the distance matrices from the Outgroup *f*_3_ results by subtracting the *f*_3_ values from 1 and we performed a classical (metric) multi-dimensional scaling (MDS) with default options from such distance matrices using the function *cmdscale* in the stats package of R [version 4.0.4 (R Core Team 2021)]. When necessary, we iteratively removed the outliers and recomputed the MDS.

## Supplementary Information

Below is the link to the electronic supplementary material.

Supplementary Figures (PDF 7504 KB)

Supplementary Table 1 (XLSX 235 KB)

Supplementary Table 2 (XLSX 98 KB)

Supplementary Table 3 (XLSX 23 KB)

Supplementary Table 4 (XLSX 1982 KB)

## Data Availability

All data used in this work are publicly available.
