# Supplementary material for: Through 40,000 years of human presence in Southern Europe: the Italian case study
Source: Hum Genet. 2021 Aug 19;140(10):1417–31. doi: 10.1007/s00439-021-02328-6 (PMC8460580; doi:10.1007/s00439-021-02328-6)
Supplement: Supplementary file 1 — Supplementary Figures (PDF 7504 KB) [file 439_2021_2328_MOESM1_ESM.pdf]

Supplementary Materials for

Through 40,000 years of human presence in Southern Europe: the Italian case study

Serena Aneli[1*], Matteo Caldon[1], Tina Saupe[2], Francesco Montinaro[2,3], Luca Pagani[1,2]

1 Department of Biology, University of Padova, Padova, Italy
2 Estonian Biocentre, Institute of Genomics, University of Tartu, Tartu, Estonia
3 Department of Biology-Genetics, University of Bari, Bari, Italy

*corresponding author. Email: serena.aneli@unipd.it

This PDF file includes:

- Supplementary Figures 1 to 5.

Other Supplementary Materials for this manuscript include the following:

- **Supplementary Table 1.** List and available information for 2,144 ancient samples used in this work.
- **Supplementary Table 2.** List of modern samples used in the Principal Component Analysis (PCA). The sheet "1240K+HO" contains the modern samples from the 1240K+HO dataset (V42.4) used to compute the principal components. The sheet "Raveane et al. 2019" contains the additional modern Italian samples from Raveane et al. (2019) that were projected, together with the ancient ones, onto the components inferred from 1240K+HO modern samples.
- **Supplementary Table 3.** List of modern samples from the 1240K dataset and from Raveane at al. (2019) used for allele frequencies computation for the twelve genetic variants under selection.
- **Supplementary Table 4.** f_3 outgroup results.

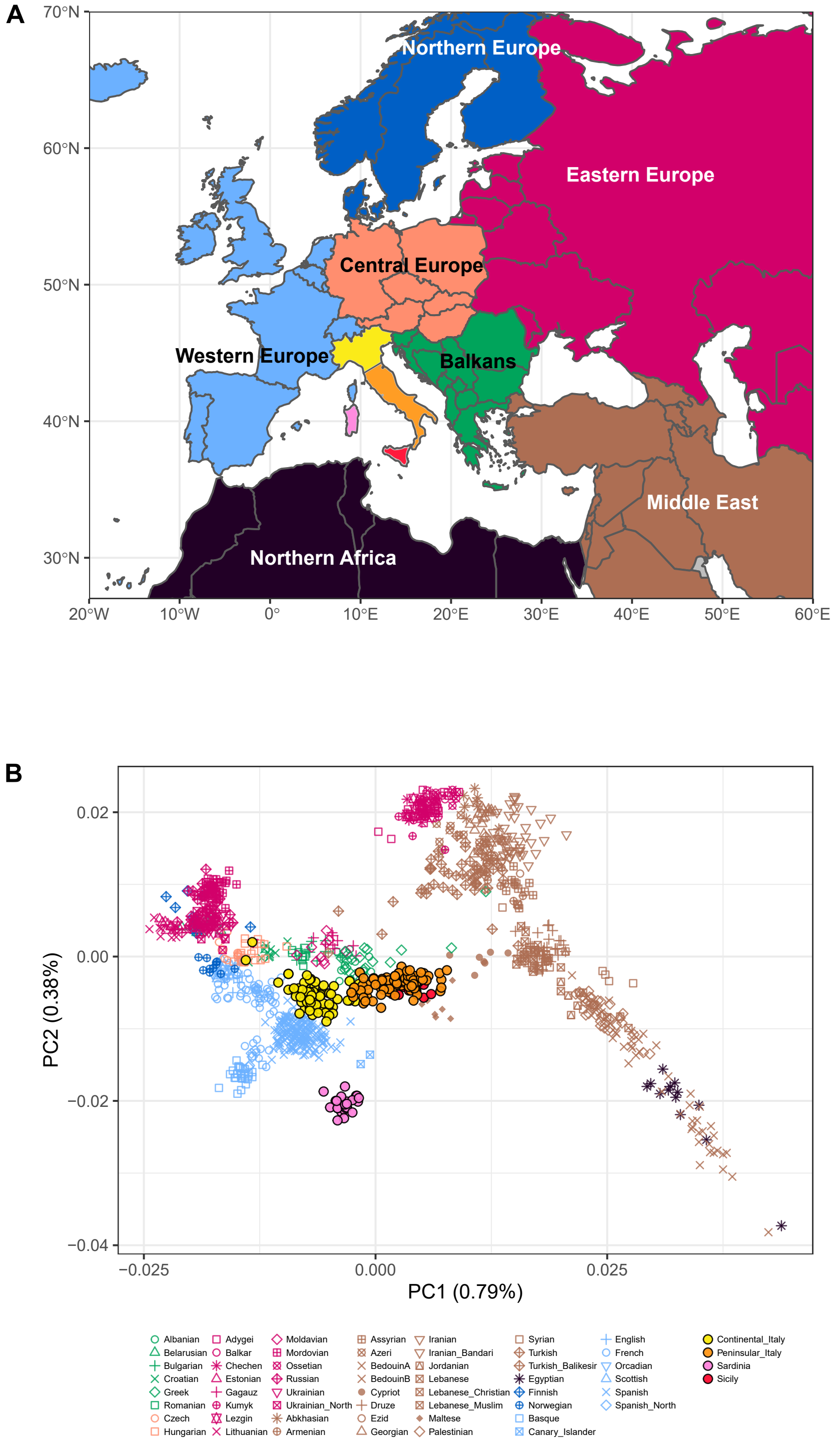

Supplementary Figure 1. Geographical location of modern samples and their genetic variability. A) Map of Western Eurasia with the indication of the macro-areas subdivisions used in the review. B) PCA of modern samples with colors and shapes corresponding to the macro-areas and the population groups, respectively.

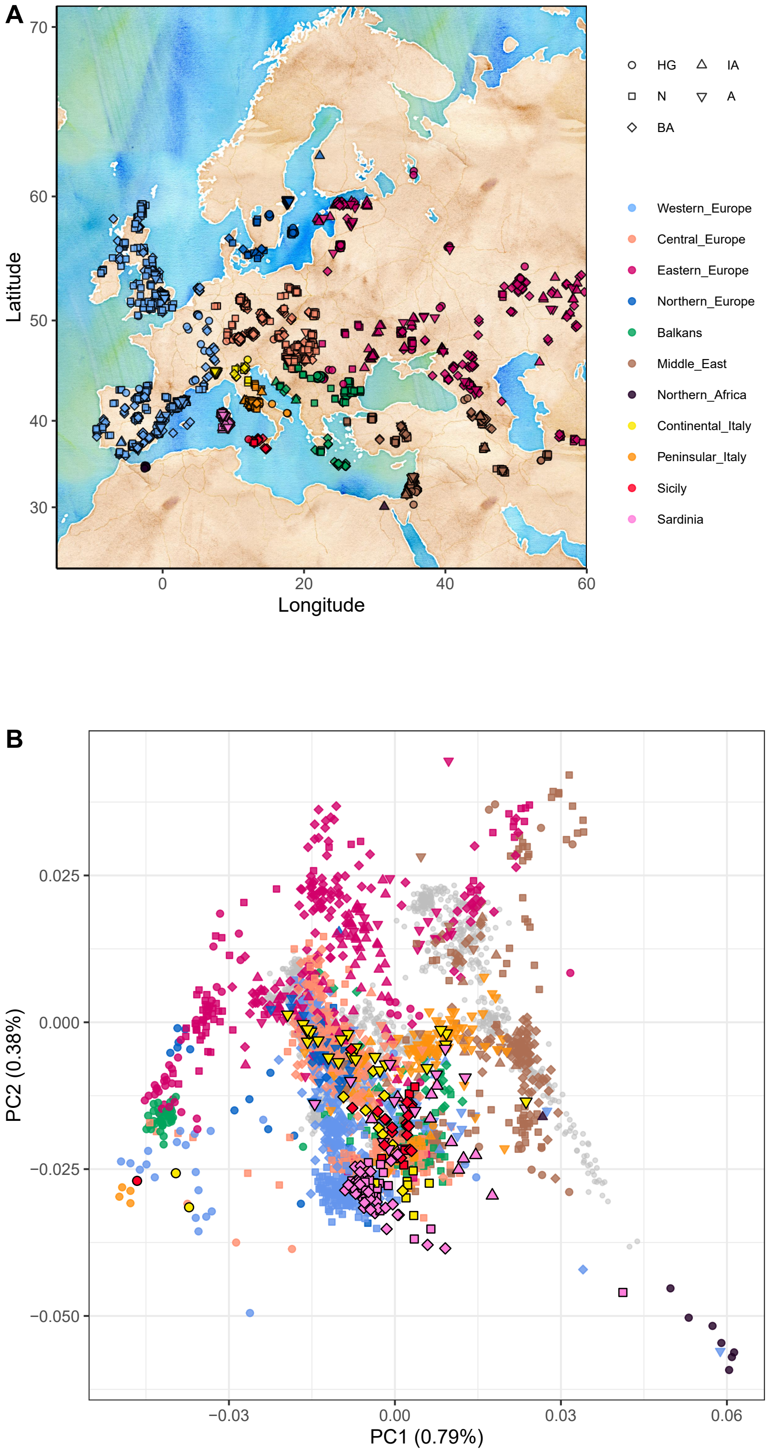

Supplementary Figure 2. Geographical location of ancient samples and their genetic variability. A) Map of Western Eurasia and geographical location of ancient samples analysed in this review. B) PCA projecting the ancient samples onto the components inferred from modern ones (in grey). Colors and shapes corresponding to the macro-areas and time layers, respectively. HG: hunter-gatherers; N: Neolithic; BA: Bronze Age; IA: Iron Age; A: Antiquity.

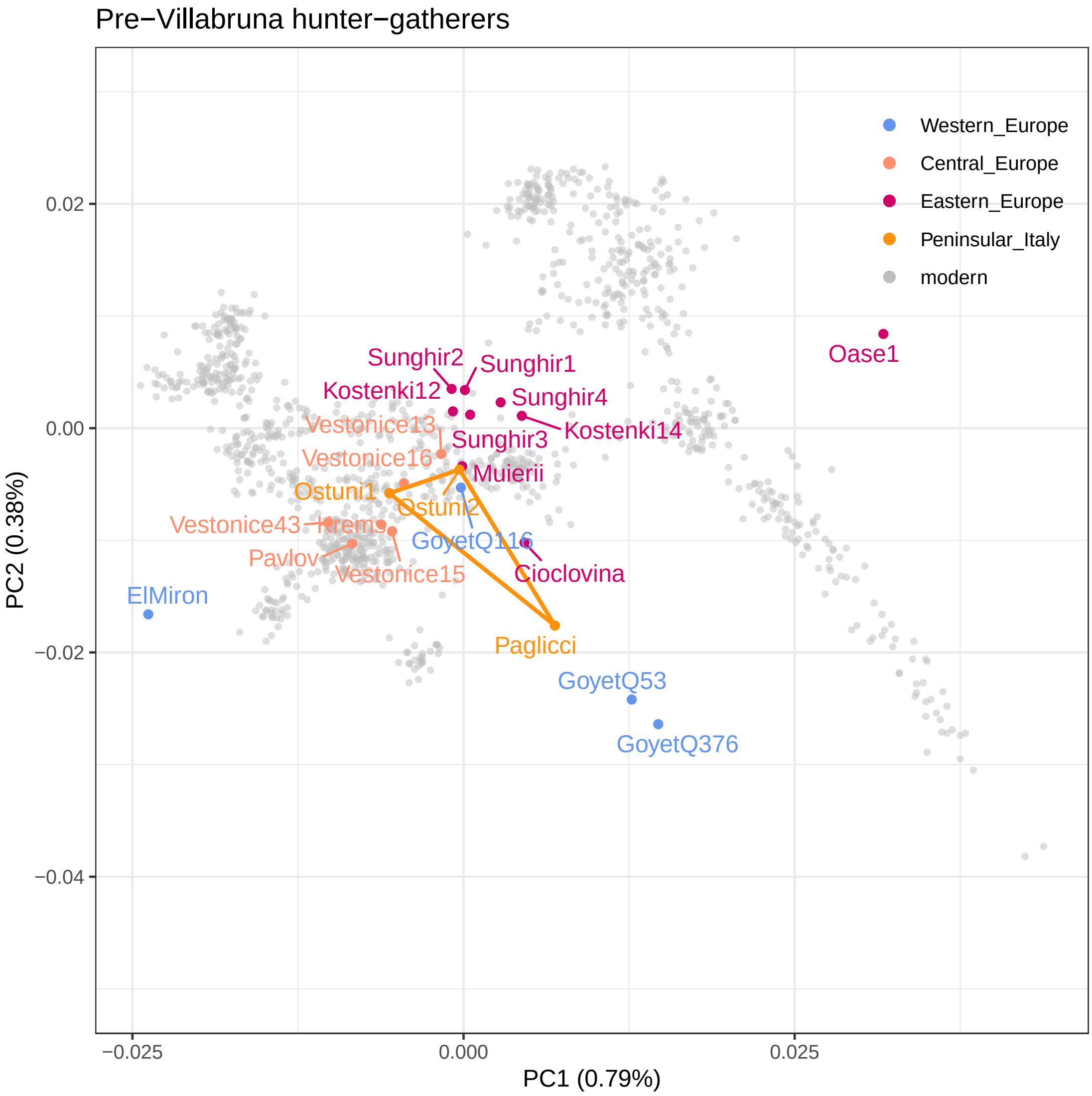

Supplementary Figure 3. PCA of Pre-Villabruna hunter-gatherers. Pre-Villabruna hunter-gatherer individuals projected onto the components inferred from modern samples (in grey). Colors correspond to the geographical area where the ancient samples had been recovered.

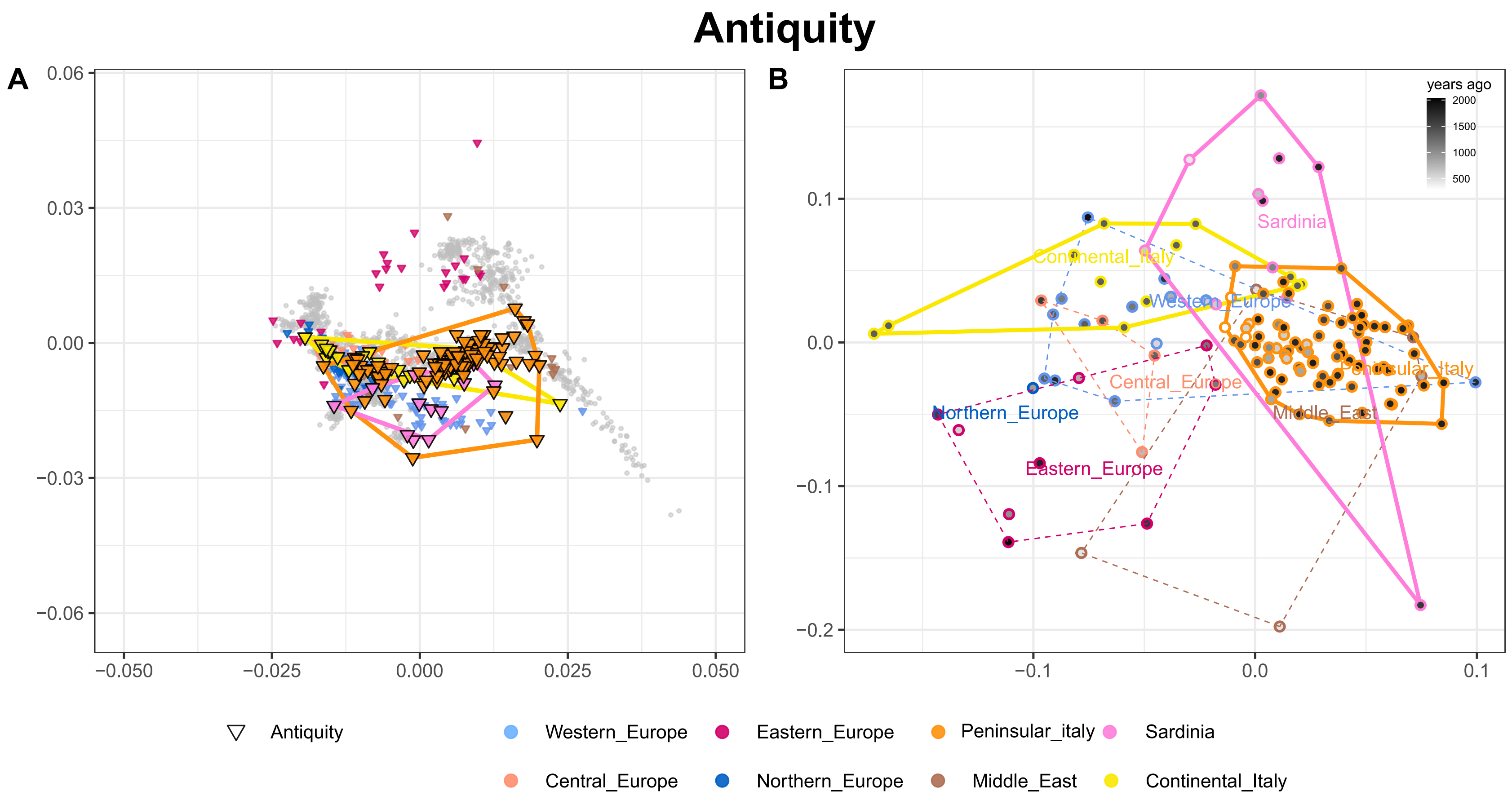

Supplementary Figure 4. Genetic analysis of individuals dated to the Antiquity period. PCA (A) and outgroup-f3 MDS (B) for the "Antiquity" time layer. Colors correspond to the geographical macro-areas assigned to the samples.

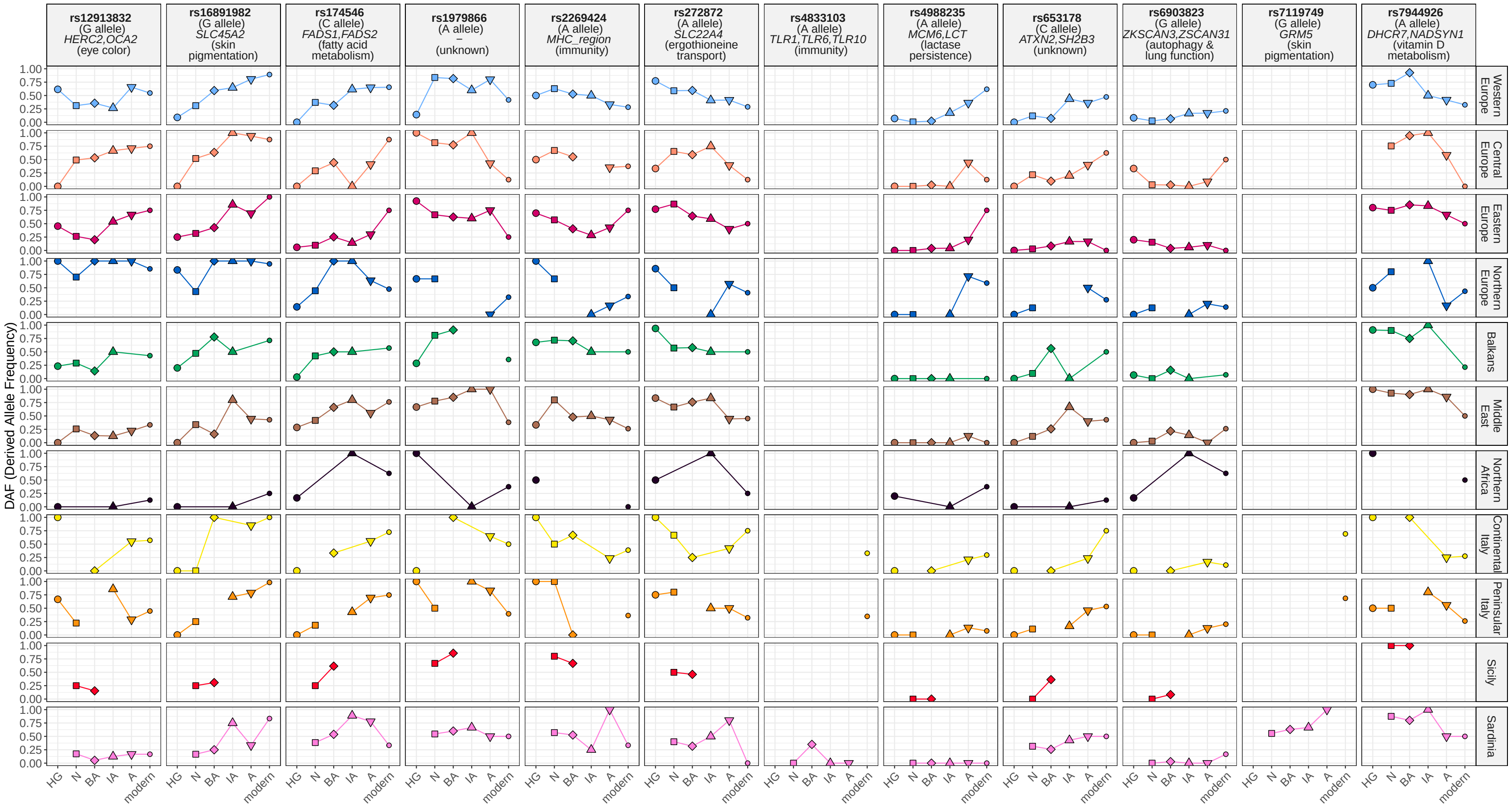

Supplementary Figure 5. Derived allele frequencies of twelve variants, reported to be significant signals of selection in literature (see Materials and Methods), across all the macro-areas and the time layers. Name of the variant (in bold), the allele for which the genetic frequencies are computed (the derived one), the gene where it falls, and the associated function are shown above each plot.
